# Standardized protocol for labor induction: a type I hybrid effectiveness-implementation trial

**DOI:** 10.1016/j.lana.2024.100956

**Published:** 2024-12-10

**Authors:** Rebecca F. Hamm, Janice Benny, Rinad S. Beidas, Knashawn H. Morales, Sindhu K. Srinivas, Samuel Parry, Lisa D. Levine

**Affiliations:** a Department of Obstetrics & Gynecology, University of Pennsylvania Perelman School of Medicine, Philadelphia, PA, USA; b Leonard Davis Institute of Health Economics, Perelman School of Medicine, University of Pennsylvania, Philadelphia, PA, USA; c Department of Medical Social Sciences, Feinberg School of Medicine, Northwestern University, Chicago, IL, USA; d Department of Biostatistics, Epidemiology and Informatics, University of Pennsylvania Perelman School of Medicine, Philadelphia, PA, USA

**Keywords:** Implementation science, Labor induction, Cesarean delivery, Maternal morbidity, Standardization, Protocols, Fidelity

## Abstract

**Background:**

Cesarean delivery remains the most common obstetrical procedure with more than 250,000 patients in the US undergoing cesarean following labor induction annually. Here, we evaluated the impact of prospectively implementing a standardized labor induction protocol on cesarean delivery rates.

**Methods:**

This multi-site type I hybrid effectiveness-implementation study compared 2 years before (PRE) and 2 years after (POST) implementation of a standardized labor induction protocol at two hospitals within the University of Pennsylvania Health System (2018–2022). The protocol included multiple components and recommended active management of labor induction, including frequent cervical examinations, amniotomy if cervical exam ≥4 cm, and interventions for labor dystocia. The primary effectiveness outcome was cesarean delivery. Secondary effectiveness outcomes included labor length, chorioamnionitis, and maternal and neonatal morbidity. The primary implementation outcome was fidelity, defined as adherence to ≥75% of the protocol components among 8 individual components that could be evaluated discretely. All data was collected via individual chart review.

**Findings:**

8509 patients were included (PRE: n = 4214, POST: n = 4295). Our population was of median age of 31 years interquartile range (IQR) [26–35], and 44.6% identified as Black, 40.1% as white, 6.9% as Asian, and 8.4% as other or unknown; 7.4% of the population identified as Latinx. There was no significant difference in cesarean delivery rate between the two time periods overall (PRE: 21.6% vs. POST: 21.8%, p = 0.85; adjusted relative risk (aRR) 0.99 95% confidence interval (CI) [0.90–1.09]). There were no significant differences in labor length, chorioamnionitis, or composite neonatal morbidity. Maternal morbidity decreased PRE to POST (PRE: 9.3% vs. POST: 6.5%, p < 0.001; aRR 0.67 95% CI [0.58–0.79]). POST-implementation, inductions with fidelity to ≥75% of protocol components increased (PRE: 52.4% vs. POST: 59.6%, p < 0.001), evidenced by more frequent cervical examinations, earlier dilation at amniotomy, and increased labor dystocia management.

**Interpretation:**

Despite increasing standardized induction management, no significant difference in cesarean delivery was found.

**Funding:**

NICHD K23HD102523.

**Research in context**

**Evidence before this study**

The sharp increase in the cesarean rate without a subsequent decrease in maternal or neonatal morbidity has raised significant concern that cesarean delivery is overused, particularly in the United States (US). Labor induction, which makes up 30% of all deliveries in the US, occurs in almost 1.2 million US women annually, is associated with a cesarean delivery rate of around 25% nationally. Components of labor induction decision-making differ significantly across and within centers, leading to the hypothesis that standardization of these processes has the potential to improve obstetric outcomes.

We searched the PubMed database from inception to April 30, 2024 for published studies pertaining to the impact of standardized labor induction protocols using the search terms: “labor induction”, “standardization”, “standardized protocols”, “induction protocol”, “cesarean delivery”, and “maternal morbidity”. Several recent studies have evaluated the impact of standardizing labor induction processes. Suresh et al. evaluated over 800 inductions in a single-site pre-post implementation study, focusing on standardizing cervical ripening and early amniotomy. Suresh et al. demonstrated decreased time to delivery of about 1.5 h without an impact on mode of delivery, maternal, or neonatal morbidity. Lutgendorf et al. performed a pre-post evaluation of approximately 1400 inductions, and demonstrated no impact on labor length, mode of delivery, or neonatal morbidity. Importantly, neither of these studies utilized the same combination of multiple evidence-based practices into a comprehensive protocol. These prior studies were both single-site, retrospective, and utilized time to delivery as the primary outcome, therefore were not powered to detect differences in mode of delivery. Furthermore, implementation was not thoroughly evaluated in prior work.

**Added value of this study**

This study is the first large, prospective type I hybrid effectiveness-implementation study of standardized protocol for labor induction utilizing individual chart review to evaluate protocol fidelity as well as critical clinical outcomes. Here, we demonstrate that implementation of a standardized protocol for labor induction was not associated with a difference in cesarean delivery rate or labor length. There is, however, a decrease in composite maternal morbidity as well as postpartum hemorrhage, when labor induction is standardized, plausibly due to a significant increase in the utilization of evidence-based recommendations for active management of induction. Importantly, when a standardized induction protocol is highly adhered to, implementation of a standardized induction protocol is associated with improved cesarean rates, reduced time to delivery, and improved maternal morbidity rates.

**Implications of all the available evidence**

This study is the first to rigorously elucidate the clinical impact of implementing a standardized labor induction protocol. Our work evaluated implementation outcomes, including protocol fidelity, demonstrating differences in adherence by component. Future research is needed to better understand the individual components of a standardized labor induction protocol that drive outcomes. Ongoing work is being performed to determine implementation strategies, mapped specifically to barriers identified in our qualitative work, with the potential to more effectively implement these recommendations.

## Introduction

The sharp increase in the cesarean rate without a subsequent decrease in maternal or neonatal morbidity has raised significant concern that cesarean delivery is overused, particularly in the United States (US).^1^ Cesarean delivery (CD) is associated with short-term morbidity such as hemorrhage, blood product transfusion, and wound complications, and has downstream effects on subsequent pregnancy outcomes, increasing risk of placental abnormalities.^2^ Analyses have demonstrated that 15–40% of cesareans may be medically unnecessary.^3^^,^^4^ Large-scale work that effectively reduces the primary cesarean rate has thus far been limited.^1^^,^^5^

The utilization of protocols to standardize care has decreased adverse outcomes in various medical fields, including in obstetrics.^6^, ^7^, ^8^, ^9^, ^10^, ^11^, ^12^, ^13^ With wide variation by site and clinician across the US, labor management practices are an ideal target for such a standardization intervention. Induction of labor (IOL), which makes up 30% of all deliveries in the US, occurs in almost 1.2 million US pregnant patients annually,^14^^,^^15^ is associated with a cesarean delivery rate of around 25% nationally. Thus, labor induction results in approximately 250,000 yearly US cesareans.^16^ In addition, with data supporting elective induction prior to 40 weeks gestation,^17^ induction rates are likely to continue to climb in coming years.^18^^,^^19^ Management of labor induction can vary even more significantly than spontaneous labor management. Components of labor induction decision-making, such as frequency of cervical examinations, utilization of artificial membrane rupture, oxytocin, intrauterine pressure catheters, and thresholds for cesarean section, differ significantly across and within centers.

Retrospective research investigations by our group compared patients enrolled in a randomized trial that utilized a standardized labor induction protocol to a concurrent observational cohort with labor induction managed at clinician discretion.^20^ We found that utilization of a standardized labor induction protocol was associated with significant reductions in composite neonatal morbidity. While cesarean rate was lower when comparing the protocol to the observational group, this finding did not reach statistical significance, and may have been limited by small sample size. Therefore, we hypothesized that standardization of labor induction had the potential to reduce cesarean delivery rates. The aim of this type I hybrid effectiveness-implementation study was to prospectively evaluate a novel method to decrease the primary cesarean rate: standardization of labor induction, in a sample with the appropriate power to detect a meaningful difference. Simultaneously, we planned to evaluate implementation outcomes related to implementation of a standardized labor induction protocol in order to prepare for scale-up and dissemination.

## Methods

**Fig. 1:**
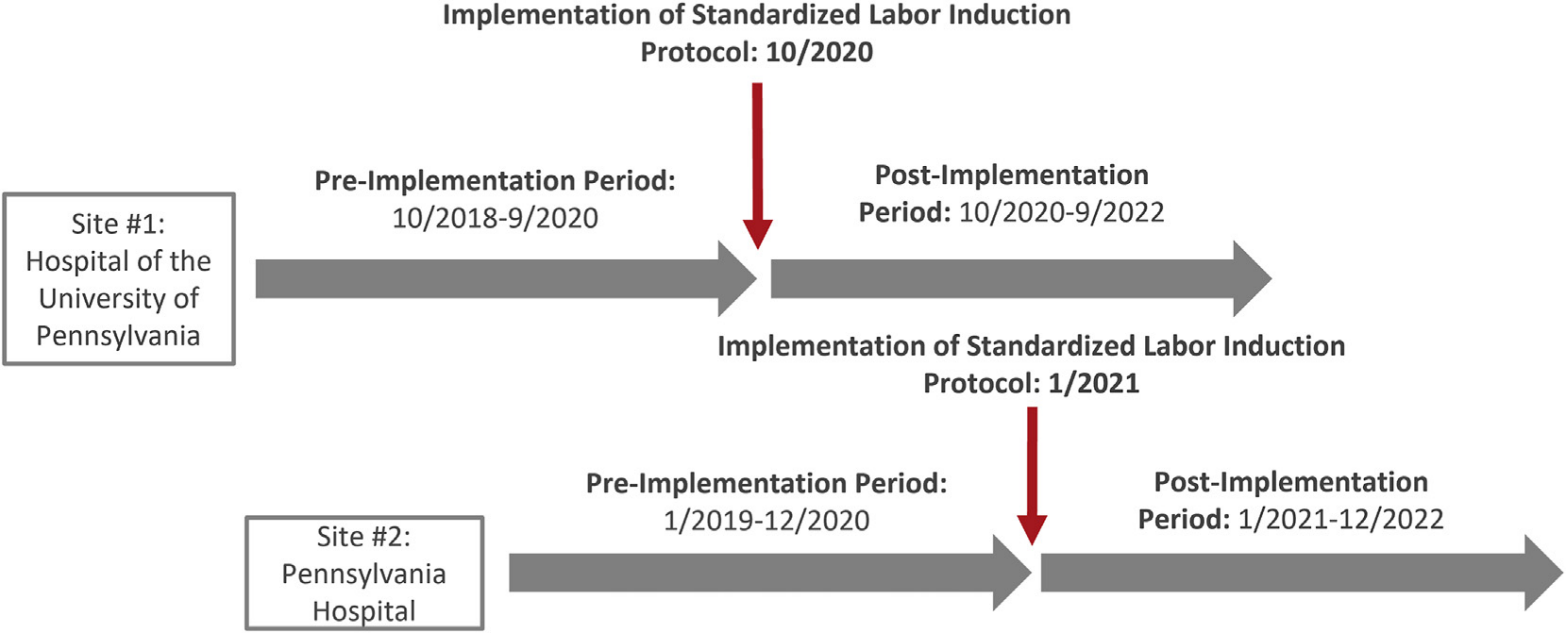
Study design.

This prospective cohort study uses a pre- (PRE) and post- (POST) implementation analysis to determine effectiveness of a standardized labor induction protocol at two sites, while simultaneously evaluating implementation outcomes (a type I hybrid effectiveness-implementation study).^21^ Implementation of a labor induction protocol occurred in a stepped approach at two separate hospitals within the University of Pennsylvania Health System. Both sites are urban hospitals with busy obstetrical services—delivery volume at Site #1 is 4100/year, while at Site #2 is 4800/year. Implementation commenced October 1, 2020 at Site #1 and January 1, 2021 at Site #2, making the PRE period October 1, 2018 to September 30, 2020 at Site #1, and January 1, 2019 to December 31, 2020 at Site #2. The POST period occurred from October 1, 2020 to September 30, 2022 at Site #1 and January 1, 2021 to December 31, 2022 at Site #2 (Fig. 1). Patients were grouped from both sites into PRE or POST implementation arms and compared PRE versus POST.

The project was approved by the University of Pennsylvania institutional review board as quality improvement with a waiver of informed consent. The Strengthening the Reporting of Observational Studies in Epidemiology (STROBE) reporting guidelines were followed in the writing of this report.^22^

### Study population

Pregnant patients were included in this study if they were undergoing a term (≥37 weeks) labor induction for any indication and met the following inclusion criteria: singleton gestation in cephalic presentation, intact membranes, and determined to require cervical ripening by their clinician. Both nulliparous and multiparous patients were included. Patients were excluded from the study if they had a prior cesarean delivery.

### Implementation of the standardized protocol

The protocol included multiple components and recommended active management of labor induction. Details of the protocols themselves are outlined in Supplemental Figure S1. Some key highlights from the protocol include: (1) Encouragement of combination cervical ripening although ultimate choice of cervical ripening method was up to the discretion of the clinician. If misoprostol was used, dosing was recommended at 25 mcg per vagina every 3 h; if a Foley balloon was utilized, it could be inflated to 30–60 cc depending on unit protocol and was removed if still in place after 12 h. (2) Hospital based oxytocin protocol was utilized (2 units per minute, increasing by 2 units per minute every 15 min as indicated, until a maximum dose of 40 units per minute). (3) Regular cervical examinations to assess for labor progress: every 2–4 h in latent labor, and every 1–2 h in active labor. (4) Interventions if labor was not progressing which included, for example, initiation of oxytocin, amniotomy, and use of intrauterine pressure catheter. (5) Amniotic membrane rupture if membranes were still intact and the cervix was ≥4-cm dilated, if clinically feasible.

Our implementation process involved 4 evidence-based implementation strategies guided by the Powell et al., 2012^23^ framework of planning, education, restructuring, and quality management. In the planning process, multidisciplinary stakeholder buy-in for the project had been obtained at both sites. Other strategies were also selected based on barriers identified in initial stakeholder meetings: (1) formal training sessions with all obstetric providers and nursing staff were held in the 3 months prior to implementation at each site, and (2) site-specific feedback regarding use of and adherence to the protocol for qualifying patients was provided to each site every 3 months throughout the POST period.

### Outcomes

The primary effectiveness outcome was cesarean delivery for any indication. Secondary maternal effectiveness outcomes included: time to delivery (defined as time from start of induction to delivery in hours), time to active labor (defined as time from start of induction to first exam at ≥6 cm or delivery if active labor was not reached), length of active labor (first exam at ≥6 cm to delivery), length of second stage, clinical chorioamnionitis, postpartum hemorrhage (estimated blood loss ≥1000 cc for any mode of delivery), and composite maternal morbidity (defined as ≥1 of the following: endometritis, blood transfusion, wound infection or separation requiring intervention, venous thromboembolism, hysterectomy, intensive care unit admission, readmission within 30 days, and death). Secondary neonatal effectiveness outcomes include composite neonatal morbidity (defined as ≥1 of the following: severe respiratory distress, culture-proven sepsis requiring antibiotic therapy, neonatal hypoxic-ischemic encephalopathy, intraventricular hemorrhage, or neonatal death) and neonatal intensive care unit (NICU) admission >48 h. Severe respiratory distress was defined as intubation and/or additional oxygen support beyond nasal cannula (i.e., continuous positive airway pressure) required outside of the delivery room.

The implementation outcomes^24^ in this study included fidelity and patient and clinician acceptability; fidelity is reported on in this paper while acceptability is reported on in a prior publication.^25^ For purposes of fidelity, we selected 8 components of the induction protocol (Supplemental Figure S1) that could be evaluated discretely in chart review. Fidelity was defined as adherence to each of the 8 specific protocol components (defined in Table 3) and was also assessed as adherence to >75% of the 8 components for which an induction was eligible. For example, if an induction ultimately was only eligible for 6 components, adherence would be to >75% of the 6 components. If a patient underwent cesarean prior to reaching active labor, they would not be eligible for adherence to the 3 components specific to active labor. Secondary labor and delivery process outcomes related to fidelity to the standardized protocol were also assessed; examples include length of cervical ripening balloon utilization (hours), number of misoprostol doses utilized, and cervical dilation at time of membrane rupture.

### Sample size

The baseline primary cesarean rate for patients undergoing labor induction at our institution was 33%. In order to demonstrate a reduction to 28%, which was the primary cesarean rate seen in preliminary data for those utilizing an induction protocol,^20^ we needed 1330 patients in each of the PRE and POST groups at a two-sided alpha of 0.05 and power of 80%. 8800 deliveries occur per year among the included units, with approximately 18% of them meeting our inclusion and exclusion criteria.^26^ Thus, a sample of 2 years PRE and 2 years POST data (3168 patients in each of the PRE and POST groups) was determined to be more than adequate to achieve our desired sample size.

### Analysis

Data from both units within the health system were pooled. All continuous variables were assessed for distribution using skewness and kurtosis tests for normality. Bivariate comparisons of demographic and pre-induction clinical characteristics by PRE and POST-implementation groups, as well as labor and delivery outcomes, were performed with Fisher's exact tests for variables where any cell n ≤ 5, and χ^2^ tests for all other categorical variables. Unpaired, 2-tailed t tests were performed for normally distributed continuous variables when homogeneity of variance was confirmed using Levene's test, and Wilcoxon rank sum tests were used for non-normally distributed continuous variables. For the primary and secondary clinical effectiveness outcomes, directed acyclic graphs (Supplemental Figure S2) were used to determine potential confounders, and variables associated with (but not affected by) both the exposure (PRE vs. POST) and the outcome independently at a p < 0.20 were assessed in modeling. Thus, body mass index (BMI), history of pre-gestational diabetes, hypertensive history, indication for induction, modified Bishop score (a standard cervical assessment scoring system),^27^ starting induction agent, and maternal age were included. Site and parity, which did not meet these criteria as possible confounders, were evaluated as interaction terms, and found not to be effect modifiers, therefore, stratified analyses for site and parity were not performed. Modified Poisson regression modeling with robust error variance was used to adjust for all confounders for binary outcomes, while Cox proportional hazards modeling was utilized for time-based variables, censoring for cesarean when evaluating length of labor. Start of labor induction defined as the time of first induction agent placement was utilized as the origin and start times for time-to-delivery hazards models. Linearity assumptions for Poisson regression modeling and proportional hazards assumptions for Cox modeling were assessed with visual plot assessments for each outcome variable.

Sensitivity analyses were performed for (1) those with Bishop ≤6 and cervical dilation ≤2 cm as there may be greater impact of the standardized protocol in a subset with less favorable cervical examination, (2) discounting the first 3 months of the PRE and POST periods (from the POST to evaluate for a learning curve with protocol use, and from the PRE to maintain the same months in the PRE and POST for analysis), and (3) comparing the entire PRE to only those patients with >75% adherence to the induction protocol in the POST period, in order to reflect an as-treated analysis. Statistical analyses were performed with Stata, version 15 (StataCorp LLC). All tests were 2 tailed, and p < 0.05 was considered statistically significant.

### Role of the funding source

This work was supported by a K23 Mentored Career Development Grant from the NICHD (K23 HD102523). The funder had no role in study design; in the collection, analysis, and interpretation of data; in the writing of the report; or in the decision to submit the paper for publication.

## Results

A total of 8509 patients met inclusion criteria across the study period; 4214 in the PRE and 4295 in the POST-implementation groups. Demographic and clinical characteristics comparing the PRE and POST periods are detailed in Table 1. Our population was 44.6% Black, 65.1% nulliparous, majority overweight or obese, and delivered at a median gestational age of 39 weeks, interquartile range (IQR) [39–40].

**Table 1:** Demographic and clinical characteristics of the pre- and post-implementation groups.

	Pre (n = 4214) n (%)	Post (n = 4295) n (%)	p-value
Site			0.76
#1	2117 (50.2)	2172 (50.6)	
#2	2097 (49.8)	2123 (49.4)	
Maternal age^a^	31 [26–34]	31 [27–35]	0.0003
Race			0.21
Black	1915 (45.4)	1881 (43.8)	
White	1666 (39.5)	1747 (40.7)	
Asian	291 (6.9)	293 (6.8)	
Other	342 (8.1)	374 (8.7)	
Ethnicity			0.076
Hispanic	292 (6.9)	341 (7.9)	
Insurance			0.87
Private	2533 (60.3)	2597 (60.5)	
Medicaid/Medicare	1667 (39.7)	1697 (39.5)	
Maternal body mass index at last prenatal visit (mg/kg^2^)^a^	31.5 [27.8–36.9]	31.8 [28.1–37.1]	0.048
Gestational or pregestational diabetes	399 (9.5)	445 (10.4)	0.17
Chronic hypertension	337 (8.0)	401 (9.3)	0.028
Nulliparity	2762 (65.5)	2781 (64.7)	0.44
Gestational age at delivery^a^	39.5 [38.6–40.4]	39.5 [38.6–40.3]	0.85
Indication for induction			<0.0001
Postdates/elective	1356 (32.2)	1351 (31.5)	
Maternal indications^b^	1460 (34.7)	1587 (37.0)	
Fetal indications^c^	870 (20.7)	747 (17.4)	
Other^d^	528 (12.5)	610 (14.2)	
Modified Bishop score^a^	2 [0–3]	2 [0–3]	<0.0001
Cervical ripening agent utilized			<0.0001
Misoprostol alone	613 (14.6)	751 (17.5)	
Cervical ripening balloon alone	163 (3.9)	129 (3.0)	
Misoprostol & ripening balloon simultaneously	2051 (48.7)	2014 (46.9)	
Oxytocin & ripening balloon simultaneously	148 (3.5)	103 (2.4)	
Misoprostol followed by ripening balloon	971 (23.0)	1090 (25.4)	
Oxytocin followed by ripening balloon	65 (1.5)	42 (1.0)	
Ripening balloon followed by misoprostol	97 (2.3)	95 (2.2)	
Ripening balloon followed by oxytocin^e^	96 (2.3)	69 (1.6)	
Other^f^	10 (0.2)	2 (0.1)	

This study sample includes all patients admitted for labor induction at either the Hospital of the University of Pennsylvania or Pennsylvania Hospital over the study period meeting inclusion and exclusion criteria for use of the standardized labor induction protocol.

a Median Interquartile range [IQR].

b Examples include: chronic hypertension, gestational hypertension, preeclampsia, diabetes, renal disease, history of venous thromboembolism, cardiac disease or other chronic medical condition where induction was recommended.

c Examples include: Oligohydramnios, intrauterine growth restriction, abnormality on fetal testing.

d Examples of “other” include: history of an intrauterine fetal demise, vaginal bleeding at term, cholestasis.

e If oxytocin was started while ripening balloon still in place.

f Other includes Cervidil and starting oxytocin then stopping and proceeding with misoprostol.

For the primary outcome, implementation of the standardized protocol for management of IOL was not significantly associated with differences in CD rate in either unadjusted or adjusted analyses (PRE: 21.6% vs. POST: 21.8%, p = 0.85; adjusted relative risk (aRR) 0.99, 95% confidence interval (CI) [0.90–1.09]) (Table 2). For assessments of time in labor, while overall labor length and length of second stage were longer in the POST period in unadjusted analyses, adjusted analyses demonstrated no significant differences (Table 2).

**Table 2:** Primary and secondary maternal and neonatal clinical effectiveness outcomes compared among the pre- and post-implementation groups of a standardized protocol for labor induction.

	Pre (n = 4214) n (%)	Post (n = 4295) n (%)	p-value	Adjusted Relative Risk (aRR) [95% Confidence Interval]^a^
**Primary outcome:** cesarean delivery	912 (21.6)	937 (21.8)	0.85	0.99 [0.90–1.09]
**Secondary maternal outcomes**				
Time to delivery (hours)^b^^,^^c^	17.4 [11.8–25.1]	18.0 [12.0–26.4]	0.012	0.97 [0.88–1.06]^d^
Length of latent labor (hours)^c^^,^^e^	14.6 [9.9–21.4]	14.9 [10.0–22.2]	0.051	0.99 [0.91–1.09]^d^
Length of active labor (hours)^c^^,^^f^^,^^g^	1.0 [0–3.0]	1.0 [0–3.2]	0.37	0.95 [0.86–1.04]^d^
Length of second stage (hours)^c^^,^^h^	0.9 [0.3–1.7]	1.0 [0.3–1.7]	0.019	0.96 [0.87–1.05]^d^
Chorioamnionitis	478 (11.3)	435 (10.1)	0.070	0.90 [0.79–1.02]
Postpartum hemorrhage	590 (14.3)	532 (12.6)	0.024	0.85 [0.76–0.96]
Composite maternal morbidity^i^	393 (9.3)	277 (6.5)	<0.0001	0.67 [0.58–0.79]
Endometritis	66 (1.6)	44 (1.0)	0.027	0.63 [0.43–0.92]
Blood product transfusion	151 (3.6)	118 (2.7)	0.028	0.75 [0.59–0.96]
Wound infection or separation	87 (2.1)	75 (1.7)	0.28	0.82 [0.60–1.12]
Venous thromboembolism^j^	4 (0.1)	5 (0.1)	0.42	–
Hysterectomy	0 (0)	0 (0)	NA	–
Intensive care unit admission^j^	14 (0.3)	8 (0.2)	0.21	–
Readmission	118 (2.8)	71 (1.7)	<0.0001	0.58 [0.43–0.78]
Death	0 (0)	0 (0)	NA	–
Primary indication for cesarean^k^			0.56	–
Non-reassuring fetal status	382 (41.9)	358 (38.2)		
Arrest of descent	137 (15.0)	135 (14.4)		
Arrest of dilation in the active phase	126 (13.8)	132 (14.1)		
Failed induction/latent phase arrest	181 (20.0)	230 (24.6)		
Maternal request	29 (3.2)	25 (2.7)		
Umbilical cord prolapse	13 (1.4)	18 (1.9)		
Other^l^	44 (4.8)	39 (4.2)		
Cervical dilation at cesarean (cm)^c^^,^^k^	5 [4–8.5]	5 [4–8]	0.92	–
Maternal length of stay postpartum (days)^c^	1.6 [1.2–1.9]	1.5 [1.1–1.9]	<0.0001	–
**Secondary neonatal outcomes**				
Composite neonatal morbidity^m^	103 (2.4)	121 (2.8)	0.28	1.18 [0.90–1.54]
NICU admission >48 h^n^^,^^o^	198 (44.1)	253 (43.9)	0.96	1.02 [0.84–1.23]
Neonatal length of stay (days)^c^	1.6 [1.2–2.0]	1.5 [1.1–2.0]	<0.0001	–

a Adjusted for body mass index, history of pregestational diabetes, hypertensive history, indication for induction, modified Bishop score, starting induction agent, and maternal age.

b Defined as time from start of induction to delivery.

c Median Interquartile range [IQR].

d Adjusted Hazard Ratio (HR) with 95% CI, censored for cesarean where applicable.

e Time from start of induction to first exam at ≥6 cm or delivery if active labor was not reached.

f First exam at ≥6 cm to delivery.

g Among those who reached active labor.

h Among those who recached second stage.

i ≥1 of the following: endometritis, blood transfusion, wound infection or separation (requiring intervention), venous thromboembolism, hysterectomy, intensive care unit admission, readmission, and death within 30 days of delivery.

j Adjusted analysis not performed due to small sample size.

k Among those who underwent cesarean.

l Example of other indications included malpresentation diagnosed during labor, worsening placental abruption, worsening preeclampsia remote from delivery, and failed operative delivery.

m Defined as ≥1 of the following: severe respiratory distress, culture-proven sepsis requiring antibiotic therapy, neonatal hypoxic-ischemic encephalopathy, intraventricular hemorrhage, or neonatal death.

n Neonatal intensive care unit (NICU).

o Among those who went to the NICU.

There were no significant associations with protocol implementation and differences in chorioamnionitis or indication for CD (Table 2). However, there was a significant reduction in both postpartum hemorrhage (PRE: 14.3% vs. POST: 12.6%, p = 0.02) and composite maternal morbidity from PRE to POST (9.3% vs. 6.5%, p < 0.0001), which remained when controlling for confounders (aRR 0.67, 95% CI [0.58–0.79]). Maternal length of stay postpartum was slightly shorter in the POST period.

With regards to secondary neonatal outcomes, there was no significant association with a difference in composite neonatal morbidity from PRE to POST-implementation in either unadjusted or adjusted analyses (PRE: 2.4% vs. POST: 2.8%, p = 0.28; aRR 1.18 95% CI [0.90–1.54]; Table 2). There was no significant association with a change in NICU admission >48 h among those infants transferred to the NICU. There was a slightly shorter neonatal length of stay in the POST-implementation period.

Table 3 includes the eight protocol components that were assessed for fidelity to the standardized labor induction protocol. When comparing PRE and POST time periods (Supplemental Figure S3), four of the eight components were more frequently performed in the POST versus the PRE time, however only 3 of those components remained significantly different by group in adjusted analyses. Specifically, more inductions were adherent to the recommendations around frequency of cervical examinations in latent labor (PRE: 35.1% vs. POST: 40.9%, p < 0.0001; aRR 1.21, 95% CI [1.12–1.29]), amniotomy if membranes were intact at the first examination at 4 cm dilation (PRE: 46.1% vs. POST: 54.5%, p < 0.0001; aRR 1.19, 95% CI [1.11–1.28]), and placement of an intrauterine pressure catheter (IUPC) for active labor dystocia (PRE: 32.1% vs. POST: 52.1%, p < 0.0001; aRR 1.59, 95% CI [1.14–2.23]) in the POST as compared to the PRE groups. Notably, another 3 of the 8 components already had adherence rates greater than 85% in the PRE-implementation period and remained as high in the POST group. Finally, there were more inductions adherent to ≥75% of the 8 protocol components for which that induction was eligible in the POST-implementation group (PRE: 52.4% vs. POST: 59.6%, p < 0.0001; aRR 1.16 95% CI [1.09–1.23]).

**Table 3:** Fidelity to 8 individual components of the labor induction protocol compared between the pre- and post-implementation.

	Pre (n = 4214) n (%)	Post (n = 4295) n (%)	p-value	Adjusted relative risk (aRR) [95% Confidence Interval]^a^
Protocol recommendation	Adherence (Fidelity)			
1. Recommendation: If cervical ripening balloon is utilized, if remains in place at 12 h after placement, remove it and initiate/continue oxytocin. *Measure of adherence: If a ripening balloon is utilized, time from placement to expulsion or removal is <12.5 h.*	3414/3590 (95.1)	3409/3542 (96.3)	0.017	1.01 [0.97–1.06]
2. If misoprostol is utilized, it should only be repeated for up to a total of 6 doses and for no >24 h. If remains in latent labor, initiate oxytocin. *Measure of adherence: If misoprostol was utilized, no more than 6 doses were given and time from placement of first misoprostol to time of placement of final misoprostol is <24 h.*	3710/3732 (99.4)	3930/3950 (99.5)	0.62	1.00 [0.96–1.05]
3. If it has been more than 6 h since misoprostol placement (whether or not cervical ripening balloon is in place), and AROM not yet feasible with no window for another misoprostol, start oxytocin. *Measure of adherence: During cervical ripening with misoprostol, there was no window >6.5 h where no active management of latent labor was undertaken. Eligible actions included placement of a cervical ripening balloon or another misoprostol, start of oxytocin, or AROM.*^b^^,^^c^	2513/3732 (67.3)	2727/3950 (69.0)	0.11	1.04 [0.99–1.10]
4. Latent labor exams should be performed: At least every 3 h if misoprostol and/or Foley being used; At least every 4 h if oxytocin is being used. *Measure of adherence: There were no gaps between latent labor cervical exams >4.5 h.*	1479 (35.1)	1758 (40.9)	<0.0001	1.21 [1.12–1.29]
5. If patient is ≥4 cm dilated and has intact membranes, recommend performing amniotomy if feasible. *Measure of adherence: If 4 cm dilation was reached with intact membranes, amniotomy was performed at that exam.*	1333/2894 (46.1)	1576/2890 (54.5)	<0.0001	1.19 [1.11–1.28]
6. Exams should be performed every 1–2 h in active labor. *Measure of adherence: There were no gaps between active labor cervical exams >2.5 h.*^d^	3156/3690 (85.5)	3229/3760 (85.9)	0.67	1.00 [0.96–1.06]
7. If there are 2 exams in active labor 2 h apart with the same cervical dilation and membranes are already ruptured, but oxytocin has not yet been started, start oxytocin. *Measure of adherence: If there are 2 exams in active labor 2 h apart with the same cervical dilation and membranes are already ruptured, but oxytocin had not yet been started, it was begun within 30 min of the 2nd exam.*	9/32 (28.1)	12/37 (32.4)	0.70	1.31 [0.49–3.52]
8. If there are 2 exams in active labor 2 h apart with the same cervical dilation and membranes are already ruptured with oxytocin already begun, place an IUPC. *Measure of adherence: If there are 2 exams in active labor 2 h apart with the same cervical dilation and membranes are already ruptured with oxytocin already begun, an IUPC was placed within 30 min of the 2nd exam.*^e^	61/190 (32.1)	98/188 (52.1)	<0.0001	1.59 [1.14–2.23]

a Adjusted for body mass index, history of pregestational diabetes, hypertensive history, indication for induction, modified Bishop score, starting induction agent, and maternal age.

b AROM, Artificial Rupture of Membranes.

c The measure was no longer assessed once either oxytocin was initiated or AROM occurred, as this was determined to be the completion of cervical ripening.

d Among those who reached active labor ≥6 cm dilation.

e IUPC, intrauterine pressure catheter.

Changes in labor process outcomes that may have occurred as a result of alterations in adherence to the labor induction protocol were also assessed (Supplemental Table S1). Implementation of the protocol was associated with decreased time a cervical ripening balloon was in place, dilation at any membrane rupture, and time to membrane rupture. There were significant increases in number of misoprostol doses utilized, maximum dose of oxytocin utilized, and IUPC utilization from PRE to POST-implementation.

In sensitivity analyses for only those with Bishop ≤6 and cervical dilation ≤2 cm (Supplemental Table S2), and discounting the first 3 months of the PRE and POST periods (Supplemental Table S3), findings were consistent with the overall cohort, without significant associations with differences in CD rate, time to delivery, chorioamnionitis, or composite neonatal morbidity. The decrease in maternal morbidity from PRE to POST also remained significant in both sensitivity analyses.

Importantly, the sensitivity analysis reflecting an “as-treated” analysis, in which inductions with <75% protocol adherence in the POST period were excluded, is shown in Table 4. Here, implementation of a standardized protocol for labor induction was associated with reduced CD rates, time to delivery, chorioamnionitis, and composite maternal morbidity. No significant association with a change in composite neonatal morbidity was seen.

**Table 4:** Sensitivity analysis of primary and selected secondary clinical effectiveness outcomes compared among the pre- and post-implementation, excluding those in the POST period with <75% protocol adherence, to reflect an as-treated approach.

	Pre (n = 4214) n (%)	Post (n = 2560) n (%)	p-value	Adjusted relative risk (aRR) [95% Confidence Interval]^a^
Cesarean delivery	912 (21.6)	453 (17.7)	<0.0001	0.84 [0.75–0.94]
Time to delivery (hours)^b^^,^^c^	17.4 [11.8–25.1]	14.3 [9.7–20.8]	<0.0001	1.52 [1.35–1.70]^d^
Chorioamnionitis	478 (11.3)	201 (7.9)	<0.0001	0.71 [0.60–0.83]
Composite maternal morbidity^e^	393 (9.3)	147 (5.7)	<0.0001	0.61 [0.50–0.74]
Composite neonatal morbidity^f^	103 (2.4)	69 (2.7)	0.52	1.17 [0.86–1.59]

a Adjusted for body mass index, history of pregestational diabetes, hypertensive history, indication for induction, modified Bishop score, starting induction agent, and maternal age.

b Defined as time from start of induction to delivery.

c Median Interquartile range [IQR].

d Adjusted Hazard Ratio (HR) with 95% CI censored for cesarean.

e ≥1 of the following: endometritis, blood transfusion, wound infection or separation (requiring intervention), venous thromboembolism, hysterectomy, intensive care unit admission, readmission, and death within 30 days of delivery.

f Defined as ≥1 of the following: severe respiratory distress, culture-proven sepsis requiring antibiotic therapy, neonatal hypoxic-ischemic encephalopathy, intraventricular hemorrhage, or neonatal death.

## Discussion

In this type I hybrid effectiveness-implementation study, implementation of a standardized protocol for labor induction was not associated with a difference in cesarean delivery rate or labor length. There was, however, a decrease in composite maternal morbidity as well as postpartum hemorrhage, plausibly due to a significant increase in the utilization of evidence-based recommendations for active management of induction.

Several recent studies have evaluated the impact of standardizing labor induction processes. Suresh et al. evaluated over 800 inductions in a single-site pre-post implementation study, focusing on standardizing cervical ripening and early amniotomy.^28^ While components of this protocol reflected some of the components evaluated in this work, Suresh et al. demonstrated decreased time to delivery of about 1.5 h without an impact on mode of delivery, maternal, or neonatal morbidity. However, time to delivery in the post-implementation period of the Suresh study was about 20 h, still nearly 3 h longer than the length of labor seen in our overall cohort. Lutgendorf et al. performed a similar pre-post evaluation of approximately 1400 inductions, and demonstrated no impact on labor length, mode of delivery, or neonatal morbidity.^29^ However, similar to our findings, their work demonstrated a significant reduction in maternal morbidity.

Our “as-treated” sensitivity analysis demonstrated that when the protocol was highly adhered to, implementation of a standardized induction protocol was associated with improved cesarean rates, reduced time to delivery, and improved maternal morbidity rates. A plausible reason we saw no significant difference in cesarean delivery in the overall study may be because both of our sites already had high compliance with active management of labor, with multiple components of the protocol demonstrating >85% adherence at baseline, potentially biasing our results towards the null. Although there were many components we were already doing well, there were additional components where there still remained significant room for improvement. Two protocol components that began at lower baselines (<65%) did not achieve statistically significant improvements: (1) if it has been more than 6 h since misoprostol placement (whether or not cervical ripening balloon is in place), and amniotomy not yet feasible with no window for another misoprostol, start oxytocin, and (2) if there are 2 exams in active labor 2 h apart with the same cervical dilation and membranes are already ruptured, but oxytocin has not yet been started, start oxytocin Even among the 3 components for which statistical improvement was made, further success could have been achieved. Some of this practice gap can be understood through our mixed-methods work with both patients [data not yet published] and clinicians,^25^ which identified barriers to implementation possibly unmet by the implementation strategies deployed in this work. For example, interviews identified that many patients were unaware of the recommended steps of labor induction prior to presenting to the labor unit. Education in the outpatient setting to prepare patients to be active consumers of the protocol could be a target for future study.

While there was no significant change in overall cesarean delivery rate, there was a significant reduction in composite maternal morbidity associated with protocol implementation in the overall cohort, which remained in all sensitivity analyses. This finding may have been due to an increased utilization of active induction management techniques as demonstrated by an increase in the individual components of the protocol. It is important to note, however, that our hospitals participated in other ongoing quality initiatives specifically aimed at reducing postpartum hemorrhage during this time period, which could have impacted our results.^30^ Importantly, our data demonstrates that standardization of active management of labor induction, including increased frequency of cervical examinations, earlier amniotomy, and higher doses of oxytocin, at the very least did not increase rates of chorioamnionitis, obstetric hemorrhage, or endometritis.

Strengths of our study includes the large sample size of over 8500 inductions across two sites, powered to assess for a difference in a critical obstetric outcome, cesarean delivery rate. As evaluating adherence to such a complex induction protocol is often difficult in large database studies, this work assessed both protocol fidelity and outcomes utilizing individual chart review. Utilizing a type I hybrid implementation-effectiveness study also allowed for a depth of understanding of where and why protocol implementation was more or less successful, contributing to our understanding of the mechanisms by which a labor induction protocol might impact outcomes. Our study is limited by its pre-post implementation design, susceptible to unmeasured bias and confounding, as well as secular trends. The fallibility of significance testing throughout this analysis is recognized, and thus our conclusions are based primarily on adjusted risk ratios. Of note, hazard ratios, as utilized throughout this work for time-related variables, suffer from an inherent selection bias.^31^ In addition, the baseline cesarean rate seen in our PRE-implementation period (22%) was substantially lower than anticipated based on our preliminary data (33%), possibly due to interval interventions targeted at cesarean reduction, and it is possible that implementing such a protocol at sites with higher cesarean rates and longer labor lengths could have greater potential to impact the outcome of cesarean delivery.

Despite the large-scale of this work, future research is needed to better understand the individual components of a standardized labor induction protocol that drive outcomes, and what characteristics of a labor unit might allow for improved implementation and clinical success. Ongoing work is being performed to determine implementation strategies, mapped specifically to barriers identified in our qualitative work, with the potential to more effectively implement these recommendations. If high adherence could be reached across patients, it is plausible that implementation of this type of protocol could have a profound effect on a large-scale. Future work should evaluate standardization of IOL in diverse settings, including community and rural hospitals, where practices may vary even further than what was seen in this work. Finally, research is ongoing to evaluate associations between standardizing labor induction practices and disparate outcomes among patients most impacted by variation in care routed in bias, such as evaluating outcomes by race, ethnicity, insurance status, and obesity class.

In summary, implementation of standardized protocol for labor induction was associated with tangible improvements in active induction management, and improvement in maternal morbidity, without a change in the overall cesarean delivery rate, labor length, or neonatal morbidity. This work also provides valuable implementation data for sites seeking to implement similar protocols.

## Contributors

RH, LL, SP, SS, KM and RB conceived of and designed this work. RH and JB collected data. All authors assisted in data interpretation. RH drafted the manuscript, which was revised by all authors. All authors have approved the final manuscript.

## Data sharing statement

Data can be shared at reasonable request to the investigative team.

## Declaration of interests

The authors declare that they have no competing interests.
