# Supplementary material for: Standardized protocol for labor induction: a type I hybrid effectiveness-implementation trial
Source: Lancet Reg Health Am. 2024 Dec 10;41:100956. doi: 10.1016/j.lana.2024.100956 (PMC11683308; doi:10.1016/j.lana.2024.100956)
Supplement: Supplemental Document [file mmc1.docx]

Title: Standardized protocol for labor induction: a type I hybrid effectiveness-implementation trial

Table of Contents:

1. Supplemental Figure 1. Figure 1a: Standardized protocol for the management of latent labor. 1b: Standardized protocol for the management of active labor.
2. Supplemental Figure 2. Casual directed acyclic graph for the relationship between the PRE and POST implementation periods and the primary outcome of cesarean delivery.
3. Supplemental Figure 3: Fidelity to 8 individual components of the labor induction protocol compared the pre- and post- implementation.
4. Supplemental Table 1: Mechanisms of effect: labor induction process outcomes as compared pre- and post- implementation of a standardized protocol for labor induction.
5. Supplemental Table 2: Sensitivity analysis of primary and selected secondary clinical effectiveness outcomes compared among the pre- and post- implementation among only those with Bishop≤6 and cervical dilation ≤2cm.
6. Supplemental Table 3: Sensitivity analysis of primary and selected secondary clinical effectiveness outcomes compared among the pre- and post- implementation discounting the first 3 months of the PRE and POST periods.

Supplemental Figure 1:

****Figure 1a: Standardized protocol for the management of latent labor. 1b: Standardized protocol for the management of active labor.

**Supplemental Figure 2:** Casual directed acyclic graph for the relationship between the PRE and POST implementation periods and the primary outcome of cesarean delivery

**
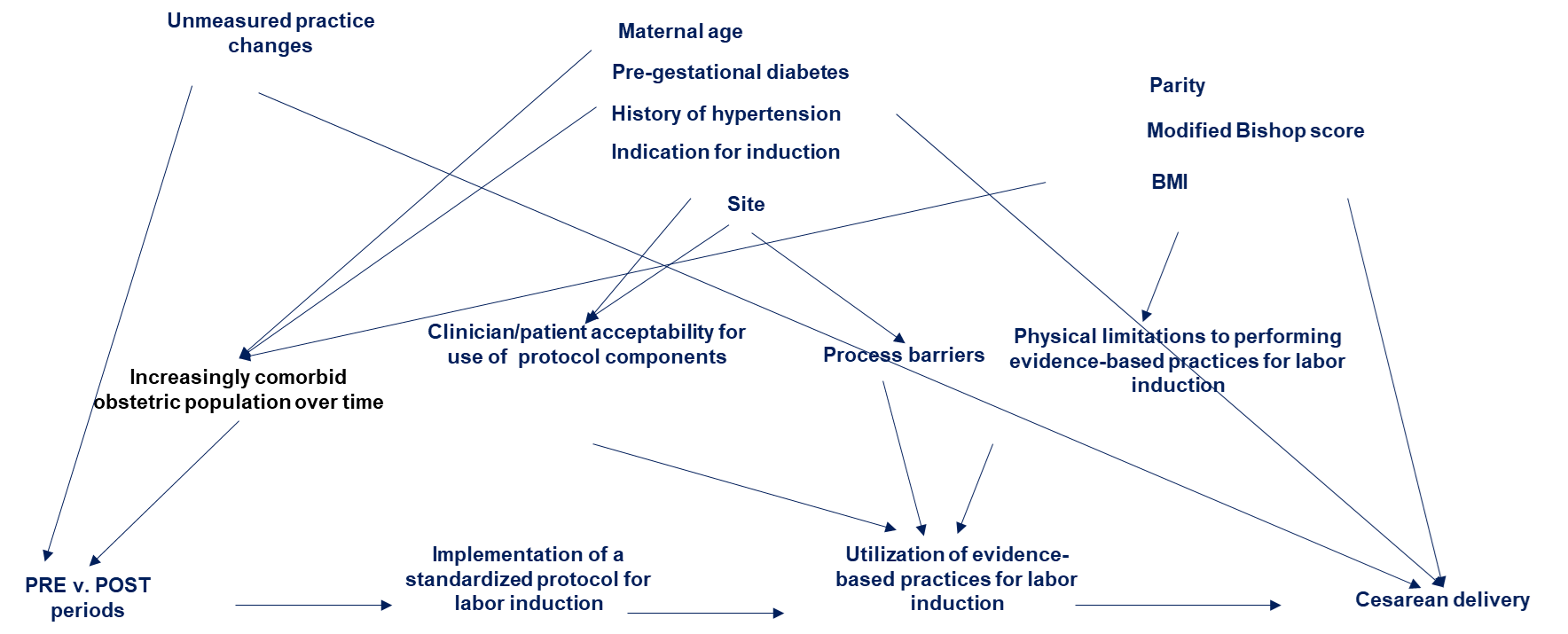
**

**Supplemental Figure 3:** Fidelity to 8 individual components of the labor induction protocol compared the pre- and post- implementation. Component numbers reflect the components as detailed in Table 3.

**
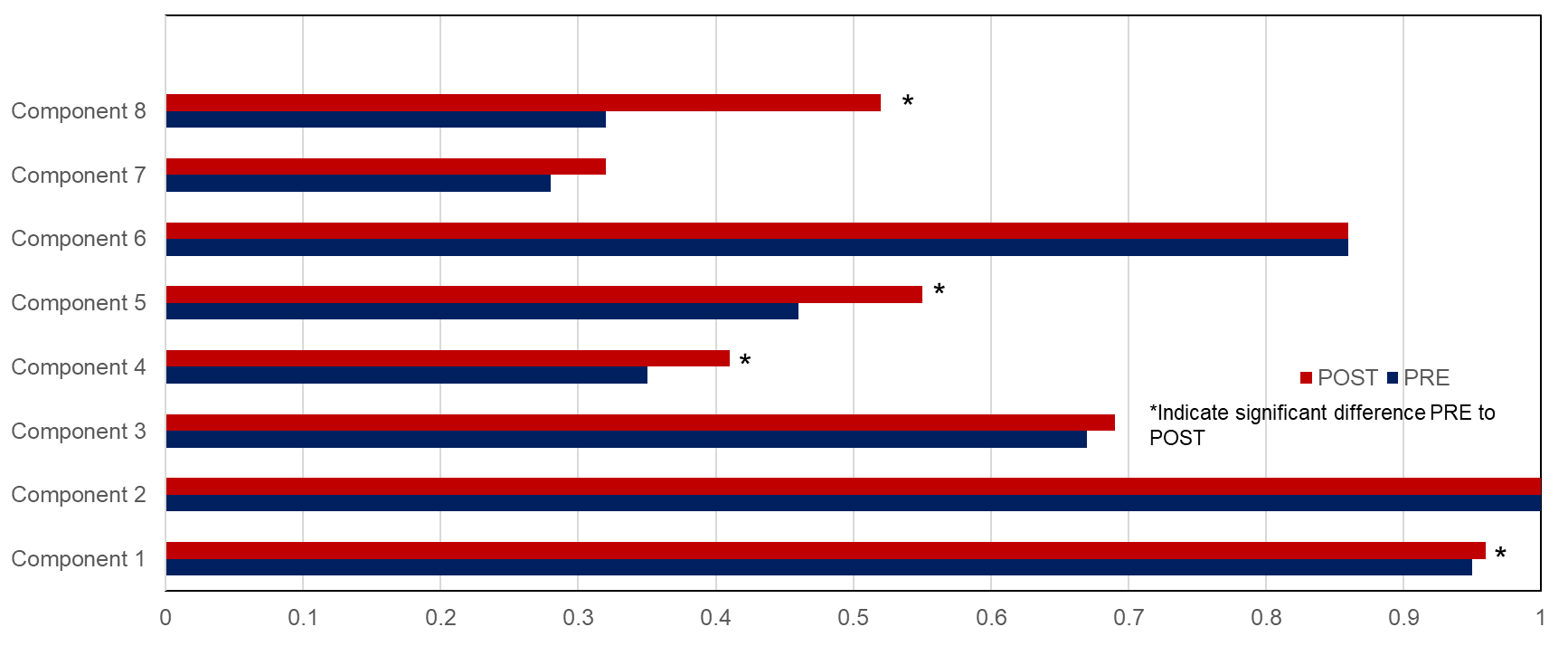
**

**Supplemental Table 1:** Mechanisms of effect: labor induction process outcomes as compared pre- and post- implementation of a standardized protocol for labor induction

|  |  | **Pre (n=4214)**  **n(%)** | **Post (n=4295)**  **n(%)** | **p-value** | **Adjusted Relative Risk (aRR) [95% Confidence Interval]^a^** |
| --- | --- | --- | --- | --- | --- |
| Length of time cervical ripening balloon in place (hours) ^b, c^ | | 4.8 [3.4-7.2] | 4.6 [3.3-7.2] | 0.051 | 1.08 [1.03-1.13] ^d^ |
| Number of misoprostol doses ^b, e^ | | 1 [1-2]; 90% 2 ^f^ | 1 [1-2]; 90% 3 ^f^ | <0.0001 | -- |
| Type of membrane rupture | |  |  | 0.073 | -- |
| *Artificial* | | 3030 (71.9) | 3170 (73.8) |  |  |
| *Spontaneous* | | 1133 (26.9) | 1088 (25.3) |  |  |
| *NA – Cesarean occurred prior* | | 50 (1.2) | 37 (0.9) |  |  |
| Dilation at membrane rupture (cm) ^b, f^ | | 4 [3.5-5] | 4 [3-5] | <0.0001 | -- |
| Dilation at AROM (cm) ^b, g,h^ | | 4 [4-5]; 95% 7.5 ^f^ | 4 [4-5] 95% 7 ^f^ | 0.0005 | -- |
| Time to membrane rupture (hours) ^b, d, g, i^ | | 9.6 [6.0-14.6] | 9.4 [5.9-14.2] | 0.22 | 1.10 [1.06-1.15] ^d^ |
| Oxytocin utilization at all | | 3473 (82.4) | 3478 (81.0) | 0.086 | 0.98 [0.94-1.03] |
| Maximum oxytocin dose ^b^ | | 10 [6-16] 90% 22 ^f^ | 10 [6-16] 90% 24 ^f^ | 0.025 | -- |
| Time to oxytocin start (hours) ^b, i^ | | 7.8 [4.9-11.9] | 8.4 [5.3-12.5] | <0.0001 | 0.98 [0.94-1.03] ^d^ |
| IUPC utilization ^j^ | | 1087 (25.8) | 1267 (29.5) | <0.0001 | 1.16 [1.07-1.26] |
| ^a^ Adjusted for body mass index, history of pregestational diabetes, hypertensive history, indication for induction, modified Bishop score, starting induction agent, and maternal age ^b^ Median[IQR]  ^c^ if cervical ripening balloon utilized ^d^ Hazard ratio (HR) ^e^ if misoprostol utilized ^f^ if median [IQR] was the same between groups, but the groups statistically differed, the 90% or 95% percentiles for that variable are shown; ^g^ If cesarean was not performed prior to membrane rupture ^h^ AROM = Artifical rupture of membranes ^i^ from induction start ^j^ IUPC = intrauterine pressure catheter | | | | | |

Supplemental Table 2: Sensitivity analysis of primary and selected secondary clinical effectiveness outcomes compared among the pre- and post- implementation among only those with Bishop≤6 and cervical dilation ≤2cm

|  |  | **Pre (n=4032)**  **n(%)** | **Post (n=4119)**  **n(%)** | **p-value** | **Adjusted Relative Risk (aRR) [95% Confidence Interval]^a^** |
| --- | --- | --- | --- | --- | --- |
| Cesarean delivery | | 757 (18.8) | 765 (18.6) | 0.81 | 0.97 [0.88-1.08] |
| Time to delivery (hours) ^b, c^ | | 17.3 [11.8-24.8] | 17.8 [11.9-25.9] | 0.076 | 0.98 [0.94-1.03] ^d^ |
| Chorioamnionitis | | 453 (11.2) | 401 (9.7) | 0.027 | 0.87 [0.76-1.00] |
| Composite maternal morbidity ^e^ | | 380 (9.4) | 274 (6.5) | <0.0001 | 0.70 [0.58-0.79] |
| Composite neonatal morbidity ^f^ | | 99 (2.5) | 118 (2.8) | 0.31 | 1.17 [0.89-1.54] |
| ^a^ Adjusted for body mass index, history of pregestational diabetes, hypertensive history, indication for induction, modified Bishop score, starting induction agent, and maternal age ^b^ defined as time from start of induction to delivery ^c^ Median[IQR] ^d^ Adjusted Hazard Ratio (HR) with 95% CI, censored for cesarean ^e^ ≥1 of the following: endometritis, blood transfusion, wound infection or separation (requiring intervention), venous thromboembolism, hysterectomy, intensive care unit admission, readmission, and death within 30 days of delivery ^f^ defined as ≥1 of the following: severe respiratory distress, culture-proven sepsis requiring antibiotic therapy, neonatal hypoxic-ischemic encephalopathy, intraventricular hemorrhage, or neonatal death | | | | | |

Supplemental Table 3: Sensitivity analysis of primary and selected secondary clinical effectiveness outcomes compared among the pre- and post- implementation discounting the first 3 months of the PRE and POST periods

|  |  | **Pre (n=3794)**  **n(%)** | **Post (n=3755)**  **n(%)** | **p-value** | **Adjusted Relative Risk (aRR) [95% Confidence Interval]^a^** |
| --- | --- | --- | --- | --- | --- |
| Cesarean delivery | | 827 (21.8) | 821 (21.9) | 0.94 | 0.98 [0.89-1.08] |
| Time to delivery (hours) ^b, c^ | | 17.4 [11.8-25.3] | 18.1 [12.1-26.7] | 0.005 | 0.95 [0.86-1.05] ^d^ |
| Chorioamnionitis | | 416 (11.0) | 377 (10.0) | 0.19 | 0.93 [0.80-1.06] |
| Composite maternal morbidity ^e^ | | 393 (9.3) | 277 (6.5) | <0.0001 | 0.67 [0.58-0.79] |
| Composite neonatal morbidity ^f^ | | 95 (2.5) | 111 (3.0) | 0.23 | 1.22 [0.92-1.62] |
| ^a^ Adjusted for body mass index, history of pregestational diabetes, hypertensive history, indication for induction, modified Bishop score, starting induction agent, and maternal age ^b^ defined as time from start of induction to delivery ^c^ Median[IQR] ^d^ Adjusted Hazard Ratio (HR) with 95% CI, censored for cesarean ^e^ ≥1 of the following: endometritis, blood transfusion, wound infection or separation (requiring intervention), venous thromboembolism, hysterectomy, intensive care unit admission, readmission, and death within 30 days of delivery defined as ≥1 of the following: severe respiratory distress, culture-proven sepsis requiring antibiotic therapy, neonatal hypoxic-ischemic encephalopathy, intraventricular hemorrhage, or neonatal death | | | | | |
